# Erectile dysfunction and associated factors among diabetic patients at, Hawassa, Southern, Ethiopia

**DOI:** 10.1186/s12902-021-00807-5

**Published:** 2021-07-01

**Authors:** Maradona Zeleke, Dejene Hailu, Deresse Daka

**Affiliations:** 1 School of Public Health, Hawassa University College of Medicine and Health Sciences, Hawassa, Ethiopia; 2 Faculty of Medicine, Hawassa University College of Medicine and Health Sciences, Hawassa, Ethiopia

**Keywords:** Erectile dysfunction, Diabetes mellitus, Prevalence, Associated factors, Hawassa

## Abstract

**Background:**

Erectile dysfunction is an inability to initiate and have a persistent erection firm enough to have satisfying sexual intercourse. The prevalence of erectile dysfunction in diabetic men is considerably high, but it is often underdiagnosed and under-managed.

**Objective:**

This study aimed to determine erectile dysfunction and associated factors among diabetic patients at, Hawassa, Southern, Ethiopia.

**Methods:**

The institution-based cross-sectional study was conducted on 352 adult male diabetic patients randomly selected from Adare general and Hawassa comprehensive specialized hospitals using a simple random sampling technique. The number of patients to be selected from each hospital was proportionally assigned based on the total population of diabetes mellitus patients following chronic care during the study period. The descriptive statistics and multiple logistic regressions (bivariate and multivariate analysis) were carried out.

**Result:**

The prevalence of erectile dysfunction was 72.2% (95%CI, 1.76–3.68). After adjusting all factors, old age, diabetes duration, drinking alcohol, and poor glycemic control had shown significant association with erectile dysfunction.

**Conclusion:**

The occurrence of erectile dysfunction in this study community is very high. Drinking alcohol, poor glycemic control, age, and duration of diabetes were predictors of erectile dysfunction in this study area. Assessment and management of erectile dysfunction in the diabetic clinic should be part of routine medical care during follow-up visits with diabetic patients. Healthcare providers should emphasize screening and treating older patients and those who have had a diabetes diagnosis for a longer duration.

**Supplementary Information:**

The online version contains supplementary material available at 10.1186/s12902-021-00807-5.

## Background

According to the national institute of health statement of consensus, erectile dysfunction is defined as an inability to achieve or maintain an erection sufficient for satisfactory sexual performance [1]. The increasing prevalence of chronic conditions, such as Diabetes mellitus (DM), chronic complications such as erectile dysfunction (ED) rapidly rising [2]. There has been an increase in the prevalence of ED in the general population, especially among diabetic patients [3, 4].

Some studies have shown that the impact of erectile dysfunction among men is both psychological and social effects such as lose of confidence in lifestyle, anxiety, depression, lose of a personal relationship, marked effects on their self-esteem, and lose of social and work activity [5].

Erectile Dysfunction (ED) is one of the major social problems causing significant distress in men. Despite the increasing difficulty in management, knowledge and understanding of factors responsible for its development are important for prevention, and care. Also, Erectile Dysfunction impairs the quality of life and is associated with depression, increased anxiety, and poor self-esteem in affected patients [6].

In 2025 about 322 million men will be affected by ED [4]. The prevalence estimates of ED in cross-sectional studies of diabetic populations range from 35 to 71% this year [4, 7].

As a study conducted in the USA, erectile dysfunction was 18.4% of the male population aged 20 years or older. However, the crude prevalence of erectile dysfunction was over 50% among men with diabetes [8]. In the study conducted in Israeli, the prevalence of ED was 57% [9], in china 90.9% [10], in India approaching 50% in both type 1 and type 2 DM [11], in Vietnam 66.9% [12], in Brazil 66.19% [13] and Iran 59.5% of type 2 DM patients [14]. According to the studies done in African countries the prevalence of ED was 71.1% in Nigeria [15], 48% in Guinea [16], 85.7% in Egypt [17], 95% in South Africa [18], 68.8% in Kenya [19], 74.1% in Ivory Coast [20], 74% in Nigeria [21], 73.9% in Zimbabwe [22], 94.7% in Nigeria, 36.0% in South Africa [23], 55.1% in Tanzania with 12.8% of participants suffering from mild dysfunction, 11.5% from moderate, and 27.9% from severe Dysfunction [3], and 69.9% Tigray Ethiopia with 32.9% suffering from mild, 31.7% moderate, and 5.2% severe erectile dysfunction [2]..

The pooled prevalence of ED among patients with diabetes in Ethiopia was 54.3% [24] with 69.9% in the Tigray region [25], 53.1% [26], and 85.5% in the Amhara region [27], 53.3% [28] and 60.4% [29] in Oromia region of Ethiopia.

The magnitude of erectile dysfunction is usually underestimated in many developing countries because of several reasons. Firstly, ED is not a life-threatening condition, thus not reported. The second is associated with stigma attached to the problem, men with the problem rarely seeking help. There is also the problem of early detection and management of factors responsible for the development of erectile dysfunction [30].

Socio-demographic factors such as Age [2, 31, 32], Occupation, Monthly income [2, 31, 33], educational status [8, 13], Marital status [33, 34], and residence [30] are major factors in ED among DM patients. Moreover, Clinical Factors such as Duration of diabetes [3, 15, 35, 36], testosterone deficiency, peripheral neuropathy, peripheral vascular disease [4], Body mass index (BMI) [33, 37], Fasting blood glucose level (FBG) [14, 31, 36], Type of DM [9], Hypertension [15, 30] and Type of anti-diabetic drugs [15], also the determinant factors of ED among DM patients. Also Behavioral or lifestyle factors such as Smoking [31, 37], not involved in physical exercise [4, 8, 33], Alcohol drinking [12, 13] and Adherence to the drug [15] is the factors that affect ED among DM patients. Therefore, this study was aimed to determine the prevalence and associated factors of ED among diabetic patients at Hawassa teaching referral hospital and Adare General Hospital in Hawassa city. There are a few reports about the ED in Ethiopia; however, there is no related study in this study area.

## Methods

### Study area and study period

The study was carried out in Hawassa city administration which is the capital of SNNPR and located 275 km south of Addis Ababa, the capital city of Ethiopia. In Hawassa city 369,908 peoples are living according to the city’s Health Department Estimation for 2017**.** The city structured by7 urban sub-cities collectively have 21 kebeles and 1 rural sub-city includes 11 kebeles. The city has 83 both public and private health institutions, one Public Referral and Teaching hospital, one Public General Hospital, 4 Private Primary Hospitals, 9 Public Health Centers, 17 Public Health Posts, and 53 Private Clinics. This study was conducted at Adare general and Hawassa University’s comprehensive specialized hospital from January 1 to 30, 2018.

### Study design

A hospital based cross-sectional study was conducted on 356 male diabetic patients attending two hospitals in the Hawassa City, Ethiopia.

### Source and study population

#### Source population

The source population constitutes all diabetic patients who are attending Adare general hospital and Hawassa comprehensive specialized hospital during the study period.

#### Study population

Randomly selected diabetic patients who are attending hospitals and fulfill inclusion criteria in the selected hospitals were the study population.

#### Study unit

All diabetic patients who are complaining of erectile dysfunction during the study period.

#### Inclusion criteria

Adult male patients age ≥ 18 years with a diagnosis of DM was included.

#### Exclusion criteria

Study participants with known secondary ED from genetic, endocrine, neurological, or surgical cause’s patients who are severely ill and with neurocognitive impairments were excluded.

#### Sample size determination

The sample size was calculated by Epi info version 7 and using a confidence level of 95%; Marginal error of 0.05; *p* = 0.669: the estimated proportion of patients with ED, as reported by a study conducted in Tigray [2]; z = the cut off value of the Normal distribution and; d = the precision required on either side of the proportion

$$ \frac{{Z_{\left(1-\frac{\alpha }{2}\right)}}^2\ast p\left(1-p\right)}{d^2} $$

$$ n=\frac{1.96^2\ast 0.699\left(1-0.699\right)}{0.05^2}=324 $$

Considering a non-response rate and non-willingness, 10% of the sample size 32 is added to the total sample size. The estimated sample size was 356 diabetic patients.

#### Sampling procedures

Two public Hospitals with chronic care services in Hawassa city, (Hawassa University comprehensive specialized hospital and Adare general hospital) both are selected purposively. Study subjects were selected from each hospital, based on proportionality and samples from each hospital were taken by using a simple random sampling technique.

#### Data collection instrument

Data on the prevalence and associated factors for erectile dysfunction among DM patients was collected by using an interviewer-administered questionnaire. Blood pressure and fasting blood sugar values measured for regular follow up services were obtained from patients chart. To assess the level of erectile dysfunction nationally validated standard questionnaire of 5 items international index of erectile function (IIEF5) test was used. The possible scores for the IIEF-5 range from 1 to 25 (one question has scores of 1–5). Patients with a score of > 22 were considered to have normal erectile function and subjects with a score of < 22 were considered to have erectile dysfunction(<7severe erectile dysfunction; 8–11moderate; 12–16 mild-to-moderate; 17–21mild) [38].

#### Data quality assurance

The data were checked for completeness, accuracy, and clarity. The quality of the data was maintained by cross-checking daily and entering, coded, and cleaned in Epi-Info version 7, a statistical software package then transported to SPSS windows version 20. The quality of data was maintained by recruiting 2 facilitators and 2 data collectors who had taken training for 2 days before data collection on how to approach study subjects using the pretested questionnaire. The data collection tool was evaluated by experts and pre-tested on 10% (34 participants) of total study participants 1 week before data collection for the consistency of understanding and completeness. During the data collection, data collectors were intensively supervised at each site. The collected data was checked out for completeness, accuracy, and clarity by investigators. This quality checking was done daily after data collection and amendments were made before the next data collection measure. Data clean-up and cross-checking were done before analysis.

#### Data management and analysis

After coding, the data was entered using EPI INFO version 7, and it was exported and analyzed by using SPSS version 20. The descriptive statistic and multiple logistic regressions were carried out to compute the relevant association. The variable with a *P*-value < 0.25 in the bivariate analysis was entered into a multivariate logistic regression model. Significant and independently associated with dependent variables were computed and variables having *p*-value < 0.05 in the multivariate regression model were considered at a 95% confidence interval.

## Result

### Socio-demographic characteristics

From 356 study participants, 352 diabetic patients were interviewed to achieve the study aim with a 98.9% response rate. The mean age of study participants was 49.14 (+SD = 13.047) years (range:18–90 years) and the mean age((+SD) for the T1DM patients was 24.8 ((+ 8.2) years. The mean duration of a DM diagnosis was 6.10 years (SD = 4.39) (range: 1–24 years). The majority 311(88.4%) were married and the educational status of the study participants was 31, 29.8, 18.8, 12.5, and 8% for college and above, high school, primary school, read and write and have no formal education respectively.

Most participants were government employees 125(35.5%), followed by merchants 78(22.2%) and a majority of the participants 274(77.8%) were from the urban and 78(22.2%) were rural residents. The mean monthly income of participants was 2003.91 + 1269.7 SD Ethiopian Birr and 19(5.4%) of participants were below 500 ETB (Table 1).

**Table 1:** Socio-demographic characteristics of participants of diabetic patients, at HUCSH and Adare General Hospital in Hawassa city southern Ethiopia 2018(*n* = 352)

Characteristics	Frequency	Percent (%)
**Educational status**		
No formal education	28	8
Read and write	45	12.78
Primary school	65	18.75
High school	105	29.8
College and above	109	30.96
**Age**		
18–25	18	5.1
26–35	35	9.9
36–45	90	25.6
46–55	102	29
> 55	107	30.4
**Occupation**		
Unemployed	48	13.6
Daily labor	5	1.4
Merchant	78	22.2
Government employee	125	35.5
Private/NGO	43	12.5
Farmer	53	15.1
**Marital status**		
Single	25	7.1
Married	311	88.4
Divorced	10	2.8
Widowed	6	1.7
**Residence**		
Rural	274	77.8
Urban	78	22.2
**Monthly income in Eth. birr**^**a**^		
< 500	19	5.4
501–1500	138	39.2
1501–2500	90	25.6
2501–3500	67	19

^a^ Ethiopian Birr

### Behavioral and clinical characteristics of participants

In this study, the alcohol consumption rate, smoking cigarette, not engaging regular exercise were 16.2, 5.7, and 71%, respectively. The mean FBS level of this study participants were 116.5 mg/dl, with 69% (*n* = 243) of participants are in the normal range (< 126 mg/dl) and 31% (*n* = 109) of participants above the normal range (≥126 mg/dl). About 93.5% (*n* = 329) had type II DM, and only 6.5% (*n* = 23) had Type I DM. The mean BMI of respondents was 22.75Kg/m^2^, while it was 19.2 75Kg/m^2^ for T1DM patients. About 72.4% (*n* = 255) of study participants were in normal range (BMI = 18.5–24.9Kg/m^2^) and 27.6% (*n* = 97) were overweight (BMI = 25–29.9Kg/m^2^).

Also, blood pressure and fasting blood glucose were obtained from the record of the DM patients. Nearly half of (48.3%) the participants were within the normal range for blood pressure (BP < 120/80 mmHg), 24% of the participants had elevated blood pressure (BP > 120–129/80 mmHg) while the rest of the participants had either Stage 1 (13.9%) or Stage 2 (13.1%) hypertension. The majority of participants (60.8%) were on oral hypoglycemic medication and while others (39.2) were using injectable medication. Three hundred and twelve (88.6%) participants were adherent to the DM drug (Table 2).

**Table 2:** Lifestyle, behavioral and clinical characteristics of participants of diabetic patients at Hawassa city, SNNPR Ethiopia, 2018 (*n* = 352)

Variables	Frequency	Percent
**Alcohol drinking**		
Yes	57	16.2
No	295	83.8
**Smoking cigar ate**		
Yes	20	5.7
No	332	94.3
**Physical exercise**		
Yes	102	29
No	250	71
**Drug adherence**		
Yes	312	88.6
No	40	11.4
**Fasting blood glucose**		
< 126	140	39.8
> 126	212	60.2
**Type of DM**		
Type I	23	6.5
Type II	329	93.5
**BMI**		
Underweight	0	0
Normal	214	60.8
Overweight	138	39.2
Obese	0	0
**BP**		
Normal	106	30.1
Elevated	30	8.6
Stage1 HTN	111	31.5
Stage2 HTN	105	29.8
**Type of hypoglycemic drug**		
Oral	214	60.8
Injectable	138	39.2
**Duration of diabetes**		
< 5 yrs	104	29.5
5–10 yrs	132	37.5
> 10 yrs	116	32.9
**Erectile dysfunction**		
Yes	254	72.2
No	98	27.8
**Category of ED (*n*** **= 254)**		
Mild	225	88.6
Moderate	19	7.5
Severe	10	3.9

### Prevalence of ED

**Fig. 1:**
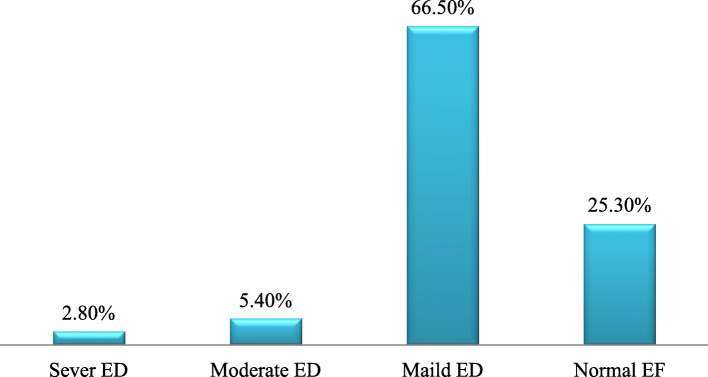
Type of severity of erectile dysfunction among DM patients attending at HURH and Adare General Hospital Hawassa, 2018

The prevalence of ED in this study was 72.2% of which 252(88.6%) had mild ED, 19(7.5%) had moderate ED and 10(3.9%) had severe ED. Only 27.8% of the patients had normal erectile function Fig. 1.

### Factors associated with ED

The bivariate logistic analysis of socio demographic characteristics with ED revealed that all socio-demographic characteristics, age (AOR = 3.2, 95%CI = 1.32–4.34, *p* = 0.04), Alcohol (AOR = 3.23, 95%CI = 1.03–10.44, *p* = 0.049), FBS (AOR = 10.3, 95%CI = 3.92–27.44, *p* = 0.00) and Diabetic duration (AOR = 17.7, 95%CI = 6.57–48.01, *p* = 0.001) showed a significant association.

In the multivariate analysis, the odds of having erectile dysfunction among diabetic patients having an age group of above 55 years was 3.2 times [AOR = 3.2, 95%CI = 1.32–4.34, *p* = 0.04)] more likely developing erectile dysfunction compared to age less than 25 years. The odds of having erectile dysfunction with a duration of diabetes > 10 years increased the risk of having erectile dysfunction [AOR = 17.7, 95%CI = 6.87–48.01, *p* = 0.001)] compared with diabetes duration of < 5 years and the diabetic duration of 5–10 years increase the risk by 2.66(AOR = 2.66, 95% CI, 1.13–6.27, *p* = 0.025) as compared with the age less than 5 years. The likelihood of erectile dysfunction among respondents who drink alcohol was increased by 3.23 times [AOR = 3.23, 95%CI: 1.03–10.44, *p* = 0.049)] as compared to their counterparts. The risk of developing erectile dysfunction among diabetics with poor glycemic control (FBS > 126 mg/dl) was increased by 10.3 times (AOR = 10.3, 95% CI, 3.92–27.44, *p* = 0.000) as compared with FBS < 126 mg/dl (Table 3).

**Table 3:** Bivariate and multivariate logistic regression of ED with predictor variables among diabetic patients in Hawassa city, Jan 2018

Variable	Erectile Dysfunction		COR(95%CI)	AOR(95%CI	***p*** value
	Yes	No			
**Age**					
15–25	3	15	1	1	
26–35	16	19	0.04(0.001–0.023)	0.238(0.026–2.168)	**0.04**
36–45	46	44	0.002(0.006–0.082)	0.274(0.026–2.84)	
45–55	84	18	0.029(0.009–0.098)	0.77(0.072–8.26)	
> 55	105	2	2.42(1.73–3.83)	3.2(1.32–4.34)*	
**Occupation**					
Unemployed	38	10	1	1	
Daily labor	1	4	0.27(0.13–0.69)	1.57(0.095–26.05)	> 0.05
Merchant	62	16	1.02(0.42–2.47)	3.06(0.299–31.33)	
Gov. employee	78	47	0.437(0.199–0.957)	1.03(0.91–11.75)	
Private/NGO	26	17	0.402(0.159–1.017)	0.76(0.071–8.26)	
Farmer	49	4	1.287(0.473–3.496)	0.6(0.04–9.01)	
**Marital status**					
Single	6	19	1	1	
Married	233	74	3.5(1.5–8)	7.3(0.937–56.84)	> 0.05
Divorced	7	3	6(1.21–29.72)	11.13(6.22–67.25)	
Widowed	8	2	4.23(2.27–6.32)	1.63(0.952–5.54)	
**Educational status**					
No formal education	25	3	1	1	
Read and write	43	2	2.3(0.48–11.31)	5.66(0.587–54.84)	> 0.05
Primary school	51	14	0.556(0.166–1.855)	1.14(0.155–8.46)	
High school	70	35	0.32(0.103–0.991)	1.57(0.196–12.71)	
College and above	65	44	0.215(0.061–0.76)	3.37(0.346–32.98)	
**Monthly income** (Eth.Birr)					
< 500 birr	15	4	1	1	
500–1500 birr	114	24	1.6(0.53–4.89)	0.71(0.097–5.32)	> 0.05
1501–2500 birr	58	32	0.21(0.045–0.99)	0.21(0.025–1.86)	
2501–3500 birr	42	25	0.56(0.18–1.75)	0.29(0.032–2.64)	
> 3500 birr	25	13	0.612(0.82–2.06)	0.30(0.03–3.03)	
**Residence**					
Urban	192	83	1	1	
Rural	62	15	1.92(1–3.69)	1.91(0.67–5.5)	> 0.05
**Alcohol**					
Yes	51	6	0.24(0.093–0.626)	3.23(1.03–10.44)*	**0.049**
No	203	92	1	1	
**Smoking**					
Yes	15	5	1.19(0.42–3.37)		
No	239	93	1	1	> 0.05
**Physical exercise**					
Yes	44	58	0.167(0.099–0.282)	0.49(0.23–1.09)	
No	210	40	1	1	> 0.05
**Adherence**					
Yes	223	89	0.785(0.369–1.67)		
No	31	9	1	1	> 0.05
**Type of DM**					
Type 1	9	14	1	1	
Type 2	245	84	5.1(2.1–12.5)	1.49(0.22–10.06)	> 0.05
**Type of hypoglycemic drug**					
Oral	133	81	1	1	
Injectable	121	17	4.04(2.3–7.06)	5.05(1.15–11.23)	> 0.05
**FBS**`					
< 126	67	73	1	1	
> 126	187	25	8.1(4.78–13.89)	10.3(3.92–27.44)**	**0.000**
**BP**			1		
Normal	35	71	4.06(1.725–9.59)	2.15(0.576–8.07)	
Elevated	20	10	14.5(7.15–29.4)	0.80(0.348–1.83)	> 0.05
Stage1 HTN	98	13	15.3(7.55–30.98)	1.49(0.42–5.29)	
Stage2 HTN	101	4	51.2(17.43–150.54)	1	
**BMI**					
Underweight	0	0			
Normal	128	86	1	1	
Overweight	126	12	7.05(0.635–1.82)		> 0.05
Obese	0	0			
**Diabetic duration**					
< 5 yrs.	45	59	1	1	
5–10 yrs.	102	30	4.45(2.54–7.82)	2.66(1.13–6.27)*	**0.025**
> 10 yrs.	107	9	15.5(7.11–34.1)	17.7(6.57–48.01)**	**0.001**

* Significant at *p*-value < 0.05. ** Significant at *p*-value < 0.001

## Discussion

In this study, the prevalence of ED among diabetic patients was 254(72.2%). This is in line with studies conducted in different parts of the world ranging between 35 and 90%. Moreover, the study finding coincides with the results from Nigeria, 71.1% [15] and the Tigray region of Ethiopia, 69.9% [2]. Also, the study conducted in Iran and Saudi Arabia showed that the prevalence of ED was 77% [14] and 80–90% [36] respectively. However, there is some discrepancy with the study conducted in Tanzania, Nigeria, Iran, Jordan, and Israel which is 55, 58, 59.5, 62, and 67%, respectively [2, 9, 32, 36]. The possible reason may be attributed to differences in the socio-cultural context of study participants, study design, and sample size.

Factors associated with erectile dysfunction are age above 55 years, duration of diabetes of above 10 years, drinking alcohol, and fasting blood glucose level > 126 mg/dl. Most studies have similar results demonstrating age and duration of diabetes, poor glycemic control, and drinking alcohol are significantly associated with ED [2, 13, 15, 30].

This study showed that older age was 3.2 times more likely to develop ED than a younger one. This is similar to the study conducted by Mebrahtu Abay [2], Mohamed AK et al. [39], Giuliano FA et al. [40], Martin. M et al. [41], Khatib F et al. [36]. This might be due to age-related physiological changes in the testicles and a decline in male sex hormones have been attributed to the increasing incidence of ED in older men.

In this study, individual engagement in an unhealthy lifestyle and behavior like drinking alcohol showed that erectile dysfunction was positively associated among DM patents. The risk is increased by 3.23 [AOR = 3.2, 95%CI = 1.03–10.44, *p* = 0.049] times more as a determinant factor compared with those who don’t drink alcohol. This result is similar to the study conducted in India and Brazil [11, 13]. Also, other researchers have presented similar results regarding alcohol as a determinant factor for ED [42–44]. Even though a few facts were evaluated in most of the studies done in other parts of the world about alcohol factors, the possible explanation might be due to alcohol abuse causing irreversible damage to nerve endings in the penis tissue. Alcohol is a depressant, and its frequent use decrease sexual desire, and make it difficult for a man to achieve erections or reach an orgasm while under the influence of alcohol. In fact, overdoing it on booze is a common cause of erectile dysfunction Moreover, intoxication can slow down or interrupt those signals between the brain and body, resulting in ED. [45]. Also, alcohol may develop stress-free lifestyles which are sedentary [45]. In this study, smoking habits did not appear as independent predictor of ED, because of low level of power of the test (64.22).

Persistently elevated blood glucose is also another statistically associated variable with erectile dysfunction in this study. This finding is consistent with the study conducted in Jordan [36] and Nigeria [15]. In this study, the risk of having ED among diabetic patients was increased by 10.3 times as compared to good glycemic controlled patients. The possible explanation for this could be poor glycemic control which is associated with nearly all microvascular complications in DM patients.

In this study, the longer duration of diabetes is significantly associated with erectile dysfunction in multivariate analysis. The risk is 17.7(CI, 6.57–48.01) times higher in duration > 10 years as compared with duration less than five. This finding is in agreement with a study from Tigray, Ethiopia, India [2, 11]. It is commonly known that many of the microvascular and macrovascular complications of DM increase with a longer duration of DM. The possible explanation of this result might be the prevalence of diabetes-related ED is mostly neurogenic and vasculogenic in etiology that increases with longer duration of DM. Moreover, as a study shown by Mohamed AK and Ahmed S, the prevalence of low testosterone was higher in patients with a longer duration of DM [39].

The average age of participants of this study is about 50 years implying that they are already at increased risk of cardiovascular diseases which could be further compounded by high prevalence of ED observed in this study. The psychosocial implication of ED will have huge impact on the quality of life of DM patients and that of their families unless it received due attention by the service providers to identify the problem at early stage and treat it.

## Limitations

This study was not measured about microvascular and macrovascular complications in DM patients. Similarly, HbA1c which could have shown glycemic control in more accurate manner, and serum testosterone levels were not measured. Therefore, this study is limited to a cross-sectional study, so that another study design is needed to determine further.

## Conclusion

In conclusion, the prevalence of ED among diabetic men is high. It increases with age, poor glycemic control, diabetic duration, and alcohol consumption. Assessment and management of ED should be part of routine medical care in diabetic follow-up clinics. Healthcare providers should openly ask men with chronic diseases, particularly those with diabetes, about symptoms of ED. An appropriate medication, sexual counseling is recommended. Further, a study with other variables is very important.

## Supplementary Information

**Additional file 1.**

## Data Availability

The data used/analyzed during the current study available from the corresponding author on reasonable request.

## Abbreviations

AOR: Adjusted odds ratio
COR: Crude odds ratio
