# Supplementary material for: Erectile dysfunction and associated factors among diabetic patients at, Hawassa, Southern, Ethiopia
Source: BMC Endocr Disord. 2021 Jul 1;21:139. doi: 10.1186/s12902-021-00807-5 (PMC8252293; doi:10.1186/s12902-021-00807-5)
Supplement: Supplementary file 1 — Additional file 1. [file 12902_2021_807_MOESM1_ESM.doc]

**Erectile dysfunction and associated factors among diabetic patients at, Hawassa, Southern, Ethiopia**

Maradona Zeleke^1^, Dejene Hailu^1^, Deresse Daka^2^*

^1^Hawassa University college of medicine and health sciences, school of public health

^2^Hawassa University college of medicine and health sciences, faculty of Medicine

*Corresponding author: [drsdk200@gmail.com](mailto:drsdk200@gmail.com)

**Annexes**
**Annex 1: Subject information sheet**

Hawassa University College of medicine and health science, school of public and environmental health

My name is ………………………………….. I am here on behalf of Maradona Zeleke, from Hawassa University College of medicine and health science, School of public and environmental health. He is conducting a research on ‘**prevalence and associated factors for erectile dysfunction among diabetics,** In Hawassa city for the partial fulfillment of master’s in public health in Hawassa University. He received permission from Hawassa university institutional review board (IRB) and the regional health bureau to conduct this study. The aim of this study is to assess ‘prevalence and associated factors for erectile dysfunction among diabetics. The study will help in providing a base line data for policy makers and other researchers on issues regarding diabetic care. You are selected randomly to participate in this study because you are eligible for this study. Your participation is purely based on your willingness. You have full right either to participate or decline to be a participant in this study. If you choose to take part in the study you may respond to all the questions or you may not answer questions you don’t want to, and have the right to stop the interview at any time. You also have the right to choose not to take part in this study. Participating in this study will not have any risk or harm. Whether you are willing to participate, refuse or decide to withdraw later, you will not be subjected to any ill treatment if you agree to participate in the study, you will be asked to answer some questions about yourself, and the interview lasts with you will take about 20-30 minutes. Any information that you provide will be kept confidential, names will not be written or specified and all the questionnaires will be coded for anonymity. No one will have access to the non-coded data except the principal investigator. Only the principal investigator will know the details and He will discard it after completing analysis. The data will not be used for purposes other than the study. Your willingness and active participation is very important for the success of this study. Contact details of principal investigator and the person to whom to contact at any time for further Explanation:

**Annex II: Informed consent**

The above information regarding my participation in the study is clear to me. I have been given a chance to ask questions and my questions have been answered to my satisfaction. My participation in this study is entirely voluntary. I understand that my records will be kept private and that I can leave the study at any time.

Respondent agree to participate?

**Yes No**

1. If yes, continue the interview
2. If no, skip to the next participant by writing reasons for his refusal.
   _____________________________________________________________________

**Informed consent Certified by:**

Respondent’s signature/thumb print____________________ Date _______________________

Interviewer: Name _________________ Signature _____________

Questionnaire ID number ________________________

Date of interview ____________ Time started __________ Time completed__________

**Result of interview:**

1. Completed 2. Respondent not available 3. Refused 4. Partially completed

Checked by: Supervisor: Name ____________________________ Signature_____________

**Annex III: Questioners**

Questionnaire prepared to assess prevalence and determinants of erectile dysfunction among DM patients

Date…………………... Study Site……………. Code……………

**Encircle only one correct response**

**Part 1: Socio demographic and economic factor**

| **No** | **Question** | **Response** | **Skip to** |
| --- | --- | --- | --- |
|  | Hospital’s Name |  |  |
| 1. | Age | ………… years |  |
| 2. | Occupation | 1. Unemployed 2. Daily laborer 3. Merchant 4. Government employee 5. Private/NGO 6. Farmer 7. Other, specify………. |  |
| 3. | Monthly income | ............. birr |  |
| 4. | Educational status | 1. Illiterate 2. Read and write 3. Primary education 4. High school 5. College and above |  |
| 5. | Marital status | 1. Single 2. Married 3. Divorced 4. Widowed |  |
| 6. | Residence | 1. Urban 2. Rural |  |

**Part 2:** Clinical factors

| **No** | **Question** | **Response** | **Skip to** |
| --- | --- | --- | --- |
| 7. | Type of DM | 1.Type 1  2.Type 2 |  |
| 8. | BMI | ……….. kg/m 2 | From the record |
| 9. | Fasting blood glucose level(FBG) | …………mg/dl | From the record |
| 10. | Duration of diabetes | …………years/months |  |
| 11. | Blood pressure | Systolic BP,,,,,,,,,,,,,mm/hg  Diastolic BP……….mm/hg |  |
| 12. | Type of hypoglycemic drug used | 1. Oral 2. Injectable |  |
| 13. | Adherence to the drug | 1. yes 2. no |  |
| 14. | If no, why? | 1. Drug shortage 2. Affordability 3. Negligence 4. Poor communication with health worker 5. Other, specify………….. |  |

**Part 3:** life style related factors

| **No** | **Question** | **Response** | **Skip to** |
| --- | --- | --- | --- |
| 15. | Do you Smoke? | 1. Yes 2. No | If no Skip to question no 17 |
| 16. | If yes For How long? | ……………. |  |
| 17. | Do you drink alcohol? | 1. Yes 2. No | If no skip to question no 19 |
| 18. | If yes For how long? | …………. |  |
| 19. | Do you do physical exercise? | 1. Yes 2. No | If no, skip to question no 21 |
| 20. | If yes, how often? | ………………. |  |

**Part 4: The five item, international index of erection function (IIEF-5) questionnaire**

| **No** | **Question** | **Response** |
| --- | --- | --- |
| 21. | How do you rate your confidence that could you get and keep an erection? | 1. very low 2. low 3. moderate 4. high 5. very high |
| 22. | When you had erection with sexual stimulation, how often were your erections hard enough for penetration? | 1. Almost never/never 2. A few times (much less than half time) 3. Some times(about half time) 4. Most time(much more than half time) 5. Almost always/always |
| 23. | During sexual intercourse, how often were you able to maintain your erection after you had penetrated (entered) your partner? | 1. Almost never/never 2. A few times (much less than half time) 3. Some times(about half time) 4. Most time(much more than half time) 5. Almost always/always |
| 24. | During sexual intercourse how difficult was it to maintain your erection to completion of intercourse? | 1. Extremely difficult 2. Very difficult 3. Difficult 4. Slightly difficult 5. Not difficult |
| 25. | When you attempted sexual intercourse, how often was it satisfactory for you? | 1. Almost never/never 2. A few times/much less than half time 3. Sometimes/about half time 4. Most time/much more than half time 5. Almost always/always |
